# Prevalence of COVID-19 Infection in Asymptomatic Cancer Patients in a District With High Prevalence of SARS-CoV-2 in Italy

**DOI:** 10.7759/cureus.13774

**Published:** 2021-03-09

**Authors:** Luigi Cavanna, Chiara Citterio, Camilla Di Nunzio, Claudia Biasini, Maria Angela Palladino, Massimo Ambroggi, Serena Madaro, Livia Bidin, Rosa Porzio, Manuela Proietto

**Affiliations:** 1 Onco-Hematology, Hospital Piacenza, Piacenza, ITA

**Keywords:** sars-cov-2, cancer, covid-19 prevalence

## Abstract

Background

Cancer patients are presumed a frail group at high risk to contract coronavirus disease (COVID-19). The aim of this study was to investigate the prevalence of SARS-CoV-2 (severe acute respiratory syndrome coronavirus 2) infection in asymptomatic cancer patients attending the outpatient clinic of a general hospital in a region with a high prevalence of SARS-CoV-2 infection (North Italy, first wave).

Methods

We retrospectively analyzed data of consecutive cancer patients attending the outpatient clinic of the oncology unit, General Hospital of Piacenza. All the patients having underlying cancer, without clinical suspicion of COVID-19, attending the outpatient clinic underwent nasopharyngeal swabs, from April 3, 2020 to June 3, 2020 and were included in this study.

Results

In a two-month period, 260 consecutive, asymptomatic (for COVID-19) cancer patients were tested for COVID-19. There were 160 women and 100 men; 218 patients were under active anticancer treatment, 32 in the diagnostic/staging phase waiting for treatment, and 10 treated with supportive care only. Ten of the 260 patients (3.85%) showed COVID-19 positivity. All but one (treated with hormone therapy) of the COVID-19 positive patients delayed anticancer treatment. The mean delay of anticancer treatment was 45.86±27.66 days (range 21-87 days), and the mean time for viral clearance was 25.7±22.68 days (range 7-79 days). All the 10 patients with COVID-19 and cancer overcame the infection, and treated patients could restart anticancer treatment.

Conclusion

Our data indicate a high prevalence of COVID-19 in cancer patients in an area with a high prevalence of SARS-CoV-2 infection. Routine COVID-19 testing of cancer patients when asymptomatic allowed an early detection, isolation, and treatment, avoiding viral spread among other frail patients and among medical/nurse staff.

## Introduction

A novel coronavirus named severe acute respiratory syndrome coronavirus 2 (SARS-CoV-2) emerged in Wuhan, China in December 2019 and has quickly spread globally [1-3].

Cancer patients are at high risk of acquiring this virus because of poor general conditions, a systemic immunosuppressive state caused by cancer itself, and/or anticancer treatment such as chemotherapy, radiation, surgery, steroids, etc. In addition, cancer patients have frequently scheduled visits to hospitals and clinics that can increase the risk of catching coronavirus disease 2019 (COVID-19) [4,5].

As reported from China, patients with cancer have a markedly elevated risk of intensive care unit (ICU) admission, intubation, or death, both for cancer patients receiving active anticancer treatment and cancer survivors [6]. Our provincial territory showed a high prevalence of COVID-19. In fact, it is very near to the epicenter of the outbreak of COVID-19 in Italy and Europe [7], so our oncologic practice has changed in response to the SARS-CoV-2 pandemic; when possible, we postponed chemotherapy treatments, surgeries were delayed, also radiotherapy was abbreviated and some treatments were switched from intravenous to oral therapies. In the oncological units, in Italy, clinical consultations, for patients who didn’t require active treatment for cancer, and follow-up were done at wider intervals of time [8-11]. According to Italian oncologists, other types of preventive measures were established to reduce virus spread; patients were subjected to triage before admission to the hospital, and more specifically, the triage screening tools used were phone calls, virtual consultations, and telemedicine [10,11]. All patients had to complete a questionnaire regarding symptoms and contact with COVID-19 patients before entering our outpatient clinic. We recently reported the first 25 hospitalized cancer patients with COVID-19 in a Western country [12], and subsequently, we described additional 51 hospitalized cancer patients with COVID-19, examining their risk factors for mortality [4]. However, data on the prevalence of COVID-19 in asymptomatic patients with cancer are fragmentary and poor [13-19]. This retrospective study was done to evaluate the prevalence of COVID-19 in an asymptomatic cancer cohort attending the outpatient clinic of a northern Italy general hospital, located in an area of north Italy with a high prevalence of COVID-19 [20].

## Materials and methods

Our oncologic department manages approximately 2,300 patients annually, including 1,200 new diagnoses. In our hospital, the first positive COVID-19 cancer patient was diagnosed on February 28, 2020. Until April 2, 2020, a COVID-19 swab was done for cancer patients without symptoms necessitating hospitalization, while it was not done for patients in the outpatient setting. Obviously, the swab test was performed on all the patients with suspected COVID-19 symptoms.

From April 03, 2020, the COVID-19 swab test was introduced for each patient who was in active anticancer treatment or about to start the treatment at the outpatient oncologic clinic. A nasopharyngeal swab was performed as previously reported [4], and briefly respiratory specimens were collected from nasopharyngeal swabs and analyzed with the reverse transcriptase-polymerase chain reaction (RT-PCR), according to the Center for Disease Control and Prevention guidelines [21]. All negative results of nasopharyngeal swabs were repeated after seven days to reduce/avoid false-negative results. Here, we analyzed data from April 3, 2020 to June 3, 2020 (two consecutive months) for COVID-19 test results in cancer patients attending our outpatient clinic.

Clinical and demographic characteristics of oncologic patients attending the oncology outpatient clinic were registered and reported.

All suspected and subsequently confirmed cases were promptly placed in isolation, when possible, at their home, in COVID-19 hotels, or hospitalized when indicated. For all patients with a positive swab or with a negative swab but COVID-19 related symptoms, a high-resolution computed tomography (CT) scan of the lungs was done. Anticancer treatment was delayed in patients with COVID-19 infection until nasopharyngeal swab was negative in two subsequent controls performed at 48 hours intervals, lungs CT scan confirmed the resolution of COVID-19 interstitial pneumonia, and patients become asymptomatic.

Data analysis

Data were collected using Microsoft Excel (Microsoft Office version 2010, Microsoft Corporation, Redmond, WA, USA). For every patient - registered with a unique recognition code - we recorded age, sex, type of cancer, stage, cancer therapy ongoing at the time of COVID-19 infection, hospitalization, radiological examinations, presence of interstitial pneumonia and laterality, antiviral therapy administered and outcome. The quantitative variables are described by mean ± standard deviation, the qualitative ones by absolute and percentage frequencies.

This retrospective study was approved by the Local Ethics Committee (Institutional Review Board approval number 494/2020/OSS*/AUSLPC) ASL of Piacenza. Informed consent was obtained from each patient.

## Results

In a two-month period, 260 cancer patients without symptoms or signs of COVID-19 attending our outpatient oncology clinic were tested for COVID-19. Among these 260 solid tumor patients, 100 (38.46%) were male and 160 female (61.54%), with a mean age of 66.06±11.17 years (range 36-91 years). Seventy-one patients had gastrointestinal cancer (27.31%), 47 breast cancer (18.08%), 41 lung cancer (15.77%), 29 gynecologic cancer (11.15%), 22 urologic cancer (8.46%), 14 head and neck cancer (5.39%), and 25 (9.62%) cancer of other sites (Table 1).

**Table 1:** Demographic and clinical data of 260 asymptomatic cancer patients swabbed for COVID-19 in an area of Italy with high prevalence of SARS-CoV-2 infection *32 patients were waiting for anticancer treatment when swabbed and 10 treated with supportive care

Variable	Tested cancer patients (n=260)	COVID-19 positive cancer patients (n=10)
Age (years), mean±ds (range)	66.06±11.17 (36-91)	69.2±7.8 (54-80)
Sex		
Male n.(%)	100 (38.46)	8(80)
Female n.(%)	160 (61.54)	2(20)
Tumor site		
Breast n.(%)	47(18.08)	0(0)
Gastro-Intestinal n.(%)	71(27.31)	5(50)
Gynecologic n.(%)	29(11.15)	0(0)
Head and Neck n.(%)	14(5.39)	0(0)
Hematologic n.(%)	11(4.23)	0(0)
Lung n.(%)	41(15.77)	2(20)
Urologic n(%)	22(8.46)	2(20)
Others n.(%)	25(9.62)	1(10)
Setting		
Neoadiuvant	10(3.85)	0(0)
Adjuvant	44(16.92)	1(10)
Metastatic	164(63.08)	9(90)
Diagnostic/staging	32(12.30)	0(0)
Supportive care	10(3.85)	0(0)
Cancer therapy		
Yes n.(%)	218(83.85)	8(80)
No n.(%)	42(16.15)	2(20)
Anticancer therapy when swabbed 218/260*		
Immunotherapy n.(%)	16(7.34)	1(12.5)
Chemotherapy n.(%)	177(81.19)	6(75)
Hormone therapy n.(%)	15(6.88)	1(12.5)
Tyrosine kinase inhibitor n. (%)	10(4.59)	0(0)

Anticancer treatment was ongoing in 218 cases (83.85%); the majority of these patients (164, 63.08%) were treated for metastatic disease, 44 (16.92%) were in the adjuvant and 10 (3.85%) in the neoadjuvant phases of treatment. Thirty-two patients (12.3%) were in the diagnostic/staging phase waiting for initiating treatment and 10 (3.85%) were treated with supportive care. For the majority of treated patients, anticancer therapy was started before the onset of the COVID-19 pandemic and it was continued during the pandemic phase. Anticancer treatment was done every week (taxol, gemcitabine, nab-paclitaxel, etc.), every two weeks (FOLFOX-4, FOLFIRI, FOLFOXIRI, etc.), or once every three weeks (FAC, etc.); other treatments were immunotherapy, tyrosine kinase inhibitors (TKI), endocrine therapy, etc.

In our series of 260 cancer patients, 10 (3.85%) showed positivity for SARS-CoV-2, so the prevalence of COVID-19 in our cohort was 3.85%. The majority of COVID-19 positive patients of our series showed a metastatic phase of cancer, and COVID-19 was diagnosed during active antitumor therapy. There were eight men and two women with a mean age of 69.2±7.8 years (range 54-80 years), while the mean age of the entire population of 260 patients that underwent nasopharyngeal swab was 66.06±11.17 years (range 36-91 years).

The types of cancer of the 10 COVID-19 positive patients were biliary tract (two patients), lung (two patients), colon (two patients), prostate (two patients), gastric (one patient), and neuroendocrine pancreatic cancer (one patient). Six patients were treated with chemotherapy, one with immunotherapy, one with hormone therapy, two with supportive care. Nine patients were treated with hydroxychloroquine-based therapy for COVID-19 at the dose previously reported [4]. All these COVID-19 positive cases underwent CT scan of the lung, nine showed interstitial pneumonia, and while asymptomatic when swabbed, these nine patients developed fever and cough after a mean of three days from nasopharyngeal swab (range 3-7 days); this is very important because if not swabbed, the nine patients could spread the virus before becoming symptomatic.

All the seven patients treated with chemotherapy (six) and immunotherapy (one) suspended anticancer treatment, and only the patient treated with hormone therapy for prostate cancer continued this treatment during COVID-19. The mean duration of anticancer suspension was 45.86±27.66 days (range 21-87 days). All the 10 patients overcame viral infection and the seven treated patients could restart their anticancer treatment.

Clinical characteristics, treatment of cancer and COVID-19, and the outcomes of the 10 patients with COVID-19 are reported in Table 2. Seven patients were managed for COVID-19 at their home in isolation and three patients were admitted to the hospital infection ward.

**Table 2:** Characteristic of 10 cancer patients positive for COVID-19 AZ: azithromycin, BP: bilateral pneumonia, CT: computed tomography, iCHE: infusional chemotherapy, oCHE: oral chemotherapy, D/C: darunavir/cobicistat, HCQ: hydroxychloroquine, HT: hormone therapy, IM: immunoterapy, NP: no pneumonia, SC: supportive care, UP: unilateral pneumonia

Patient	Age (years)	Sex	Site of cancer/stage	Cancer therapy	COVID-19 therapy	CT scan	Outcome
01	69	f	Colon/IV	iCHE	HCQ + AZ	BP	alive
02	65	m	Prostate/IV	iCHE	HCQ	BP	alive
03	64	m	Lung/IV	IM	HCQ+ D/C + AZ	BP	alive
04	69	m	Prostate/I	HT	HCQ	UP	alive
05	76	m	Biliary tract/IV	oCHE	HCQ+ D/C + AZ	BP	alive
06	74	m	Biliary tract/IV	iCHE	HCQ + AZ	BP	alive
07	80	m	Colon/IV	SC	no	NP	alive
08	54	m	Neuroendocrine pancreatic cancer/IV	iCHE	HCQ+ D/C + AZ+augmentin	UP	alive
09	64	f	Gastric/IV	SC	HCQ+ D/C + AZ+ enoxaparina	BP	alive
10	77	m	Lung/IV	iCHE	piperacilina/tazobactam + HCQ+ enoxaparina	BP	alive

## Discussion

Patients with cancer are generally presumed to have a major risk of developing infectious complications. Consequently, control measures of COVID-19 transmission are very important. Indeed, in clinical routine, asymptomatic carriers are the most challenging patients: they can infect other patients and medical/nurses staff, and they may have a severe course of COVID-19 when diagnosed with the infection while in treatment with immunosuppressive cancer therapies. It must be emphasized that the reported sensitivity of PCR of nasopharyngeal swab ranges from 80% to 54% [22,23], and it is well known that a single test can be false-negative when the infection risk exists. So in our program, the test was repeated after seven days to reduce the risk of false-negative results. In our series, we registered a 3.85% (10/260) pre or asymptomatic COVID-19 prevalence in patients with cancer.

Our data show a higher infection rate when compared with other reported studies: Fong D et al. [13] reported a prevalence of 1.8% in a tertiary care hospital in northern Italy; Marschner S et al. [14] reported an infection rate of 0.72% in Munich (Germany); Berghoff AS et al. [15] reported a low rate of detectable SARS-CoV-2 (0.4%) in a large cohort of 1,016 consecutive cancer patients in Vienna (Austria); Yu J et al. [16] reported 12 consecutive cases of COVID-19 pneumonia in 1,524 patients with cancer (0.79%) in Wuhan (China); Aznab M [17] reported three cases of COVID-19 in 279 patients (1.08%) with solid tumors and hematologic malignances in Kermanshah (Iran); Moss C et al. [18] reported nine positive cases amongh 654 cancer patients (1.38%) that were swabbed for COVID-19 in London. More recently, Aschele C et al. [19], in a large multi-institutional retrospective study in Italy, reported the incidence of COVID-19 among 59,989 patients undergoing active antitumor treatment, between January 15, 2020 and May 4, 2020: 406 (0.68%) developed COVID-19 and 77% required hospitalization. The higher infection rate (3.85%) of COVID-19 in cancer patients reported in our study might result in the highest prevalence of COVID-19 in our district: our hospital is very near the epicenter of the outbreak of COVID-19 in Italy end Europe (10 minutes by car), and the catastrophic nature of Lombardy’s outbreak has been widely published [24] in addiction, to avoid false-negative results, we performed two nasopharyngeal swab test for each patient. On the other hand, in a similar study performed in the USA, Ning et al. [25] reported a higher infection rate of 5.8%; however, in this study, there was a high percentage (55%) of symptomatic patients, while our cases were asymptomatic. In addition, a meta-analysis of studies in China reported an infection rate of COVID-19 of 2.59% in cancer patients [26].

In our 10 patients who had a confirmed COVID-19 diagnosis, delay of therapy was performed; only the patient treated with hormone therapy for prostate cancer continued his treatment. The mean length of treatment delay was six weeks, ranging from three to 12 weeks. It must be emphasized that all the patients whose treatment administration was delayed had been on therapy for at least three months before the delay and their cancer was considered to be under control. However, the mean duration of delays in cancer treatment was longer in our series when compared with other reports: Wu et al [27], in their study performed in California, USA, reported a delay in treatment with a median duration of 21 days with a range of 5-112 days, and the reasons for delays were multiple, such as the need for evidence of COVID-19 clearance for patients prior to being readmitted to the outpatient's clinic. Five of our 10 COVID-19-infected patients prolonged the delay of cancer treatment because of persistent positive nasopharyngeal swabs, while they overcame clinically and radiologically the infection. The viral clearance was achieved in a mean of 25.7±22.68 days (range 7-79 days). This time period is much longer when compared with the time period of viral clearance in non-cancer patients [28].

Our study shows some peculiarity when compared with other reports [13-19]: 1) patients performed two swabs at a week's interval, 2) we reported the treatment of COVID-19 in cancer patients, which is lacking in other studies, 3) our patients treated with chemo or immune therapy delayed their cancer treatment until complete recovery was recorded with a negative of swab test in two consecutive controls. However we are aware of some limitations of our study: it is retrospective, we cannot exclude false-negative results, and the sample size was not large.

## Conclusions

We believe that SARS-CoV-2 infection rates will decrease since vaccination is ongoing, however, our results suggest that routine COVID-19 testing for patients with cancer in outpatients clinic should be done to detect asymptomatic viral carriers and to avoid viral spread among fragile and vulnerable populations like cancer patients and among medical/nurse staff. The prevalence of COVID-19 positivity in cancer patients is related to its prevalence in the general population of the same area, which was very high in our district.
